# Phenylalanine Promotes Biofilm Formation of *Meyerozyma caribbica* to Improve Biocontrol Efficacy against Jujube Black Spot Rot

**DOI:** 10.3390/jof8121313

**Published:** 2022-12-17

**Authors:** Qian Deng, Xingmeng Lei, Hongyan Zhang, Lili Deng, Lanhua Yi, Kaifang Zeng

**Affiliations:** 1 College of Food Science, Southwest University, Chongqing 400715, China; 2 Food Storage and Logistics Research Center, Southwest University, Chongqing 400715, China; 3 Chongqing Key Laboratory of Speciality Food Co-Built by Sichuan and Chongqing, Chongqing 400715, China

**Keywords:** *Meyerozyma caribbica*, jujube, black spot rot, biofilm formation, phenylalanine

## Abstract

During storage and transportation after harvest, the jujube fruit is susceptible to black spot rot, which is caused by *Alternaria alternata*. The present study aimed to evaluate the effectiveness of the yeast *Meyerozyma caribbica* in controlling *A. alternata* in postharvest jujube fruits, and to explore the biofilm formation mechanism. The results showed that *M. caribbica* treatment significantly reduced the *A. alternata* decay in jujube fruits. *M. caribbica* could rapidly colonize jujube fruit wounds, adhering tightly to hyphae of *A. alternata*, and accompanied by the production of extracellular secretions. In *in vitro* experiments, we identified that *M. caribbica* adhered to polystyrene plates, indicating a strong biofilm-forming ability. Furthermore, we demonstrated that *M. caribbica* can secrete phenylethanol, a quorum sensing molecule which can affect biofilm development. Phenylalanine (a precursor substance for phenylethanol synthesis) enhanced the secretion of phenylethanol and promoted the formation of *M. caribbica* biofilms. Meanwhile, phenylalanine enhanced the biological control performance of *M. caribbica* against jujube black spot rot. Our study provided new insights that enhance the biological control performance of antagonistic yeast.

## 1. Introduction

The jujube fruit is a nutrient-rich fruit and a functional food that is widely appreciated by consumers for its health benefits [1]. However, fresh jujube fruit is highly susceptible to pathogenic fungi, and black spot rot caused by *Alternaria alternata* can seriously damage the commercial value of fresh jujube [2]. Chemical fungicide-induced pathogen resistance, fungicide residues, and toxicological problems have increased the urgent need for safer alternative strategies [3]. Due to their ability to manage postharvest infections without producing toxins, antagonistic yeasts are recognized as commercially viable biocontrol agents. The yeasts *Rhodosporidium paludigenum*, *Metschnikowia pulcherrima*, and *Cryptococcus laurentii* were effective in inhibiting *A. alternata* infection in postharvest jujube [4,5,6]. Antagonistic yeasts resist pathogen infection through mechanisms such as competition for nutrients and space, parasitism, secretion of antimicrobial substances, and induction of fruit resistance. Among these, the primary antagonistic mechanism of yeast is recognized as competition with pathogens for the limited nutrients and physical space in the host [7]. Antagonistic yeasts successfully compete for limited nutrient factors in the host environment and, thus, colonize rapidly. In addition, yeasts can also limit the growth of pathogens by adhering to fruit wounds and occupying limited space. Adhesion usually occurs during the formation of yeast biofilms, a specific mechanism by which yeast competes for space [8].

Biofilm formation is an effective mechanism of action for the management of postharvest diseases using antagonistic yeast [9]. For example, *C. laurentii* competed for nutrients and space with *Colletotrichum gloeosporioides* via adhesion and biofilm formation [10]. The biofilm of *Pichia kudriavzevii* exhibited better biological control than that of yeast in controlling gray mold and anthracnose in pear fruit caused by *B. cinerea* and *C. gloeosporioides* [11]. Biofilms are dense structural networks formed by small molecules (e.g., proteins, nucleic acids, polysaccharides) secreted by microorganisms during growth [12]. The formation of biofilms depends on quorum sensing (QS) between microorganisms. Microorganisms achieve cell-to-cell communication by secreting substances called “quorum sensing molecules (QSMs)” [13,14]. When QSM concentrations in the microbial community reach a certain threshold, they promote the expression of specific genes and are involved in the regulation of population behavior [15].

QSMs such as tryptophol, farnesol, and phenylethanol are commonly reported signaling molecules that perform critical roles in fungal morphological transformation and biofilm growth [16]. Among these QSMs, phenylethanol was able to affect the filamentous growth and adhesion of *Kloeckera apiculata* in citrus fruit [17]. In addition, phenylethanol was able to induce the biofilm formation of *Debaryomyces nepalensis*, which contributed to its adhesion on the jujube fruit and, as a result, protected the fruit from *A. alternata* infection [18]. Phenylethanol promoted the expression of *FLO11*, which was responsible for encoding the flocculation protein, through a tpk2p-dependent mechanism. This conferred adhesion properties to yeast and contributed to invasive cell growth, and the production of pseudohyphae and biofilm formation [19,20]. However, using phenylethanol is limited in some ways. Phenylethanol produced by chemical synthesis is limited in its application due to safety and environmental issues, while natural phenylethanol isolated from plants is limited due to its high price [21]. The synthesis of phenylethanol by microorganisms is a safe and nontoxic method.

Phenylalanine (Phe), as a precursor substance for phenylethanol, is often used in microbial fermentation to produce natural phenylethanol, which is safer than chemical synthesis [22]. Phe is relatively inexpensive and widely regarded as safe. In addition, these amino acids have an important role in the yeast culture process, and some functional amino acids are able to enhance the survival of yeast cells in extreme environments [23]. For instance, proline is used as a stress protector to enhance the resistance of industrial yeast [24], and it also promotes biofilm formation in *M. citriensis* [25]. This may be related to proline raising the content of pulcherrimin, which is a potential signaling molecule that influences the biofilm development of *M. citriensis*.

The yeast *M. caribbica* is considered to be a potential probiotic strain [26], which is widely used in food fermentation, such as functional beverages, coffee, or fruit wines [27,28]. In addition, previous studies have demonstrated that *M. caribbica* is an effective class of biocontrol agent. It has exhibited an excellent biocontrol performance in controlling the development of postharvest diseases in fruits, including mango, kiwifruit, and passion fruit [29,30]. However, the effectiveness of *M. caribbica* in biologically controlling *A. alternata* on jujube fruits is not clear, nor are the effects of *M. caribbica* biofilm formation for disease control. Although several studies have suggested the contribution of phenylethanol to yeast biofilm development, its role in *M. caribbica* biofilm formation is still unclear. It is worth investigating how Phe affects the development of *M. caribbica* biofilms and its bioprotective efficacy on jujube. Thus, the present study aimed to investigate (1) the biocontrol performance of *M. caribbica* in the control of *A. alternata* on jujube fruits, (2) the capacity of *M. caribbica* to form biofilms, (3) the biofilm formation mechanism in *M. caribbica* and its possible QSM, and (4) the influence of Phe on biofilm formation and the biocontrol efficacy of *M. caribbica* for jujube black spot rot.

## 2. Materials and Methods

### 2.1. Yeast, Pathogen, and Jujube Fruit

The yeast *M. caribbica* (KC422423.1) was preserved in our laboratory and was stored in 30% glycerol at −80 °C. It was identified by the DNA sequence of the internally transcribed spacer region. The sequences were BLASTed against the NCBI database (http://www.ncbi.nlm.nih.gov (accessed on 22 October 2021)). *M. caribbica* was grown on a NYDA medium (8 g beef paste, 5 g yeast paste, 10 g dextrose, 10 g agar, and 1000 mL distilled water; Aoboxing, Beijing, China) for 48 h before use, and it was then inoculated into the NYDB broth (NYDA without agar) to be cultured in a shaker incubator (200 rpm, 28 °C) for 16 h. Fresh *M. caribbica* cells were harvested after centrifugation (5000× *g*, 5 min) and washed twice with sterile distilled water (SDW). The *M. caribbica* cell suspension was adjusted to the desired test concentration using SDW (1 × 10^8^ cells/mL).

The fungal pathogen *A. alternata* was obtained by isolation and identification from decayed jujube fruit. *A. alternata* was cultured on the PDA medium (100 g fresh potato, 10 g glucose, 10 g agar, and 500 mL distilled water; Chronchem, Qionglai, Sichuan, China) at 25 °C. After 7 days of incubation, an *A. alternata* spore suspension was collected with SDW and then adjusted to the proper concentration (1 × 10^6^ spores/mL).

Jujube (Zizyphus jujuba cv. Dongzao) fruits were uniform in shape and color, free from both infestation and mechanical injury, and purchased from the market (Beibei, Chongqing, China). Before the experiments, the fruit was cleaned with 2% sodium hypochlorite for 2 min, rinsed in SDW, and air-dried until use.

### 2.2. Effect of M. caribbica on the Biological Control of A. alternata on Jujube Fruit

A sterile punch was used to make a 3 × 3 mm wound even on both equatorial sides of the jujube. An injection of 20 μL of *M. caribbica* cell suspension was applied to the injury of each jujube fruit, and sterile water was used as a control. After 4 h, an additional suspension of 10 μL of *A. alternata* spores was injected into each wound. Jujube fruits were air-dried before being placed in plastic baskets that maintained a stable temperature and humidity (25 °C, 90%). The detection of disease incidence (DI) and lesion diameter (LD), which define the disease progress in jujube fruit, were recorded every two days. Measurements of DI and LD were performed in accordance with the method of Liu et al. [31]. Each treatment was repeated three times, with ten fruits each time, and the entire assay was replicated twice.

### 2.3. Dynamics of M. caribbica Colonization on Jujube Wounds

Wounds were made in the fruit equator as described in Section 2.2. A 20 μL cell suspension of *M. caribbica* was incubated on the injury of each jujube. All jujube fruits were stored at 25 °C after inoculation. Jujube sample tissue was extracted from wounds on days 0 (2 h after inoculation), 1, 2, 3, 4, 5, 6, 7, 8, and 9 for population monitoring. The tissues (10 mm diameter) from the fruit wounds were macerated in 10 mL of phosphate-buffered saline (PBS, pH 7.2) and diluted 10-fold according to the sequence. The appropriate diluted yeast solution was evenly distributed on the NYDA medium. All plates were cultivated under 28 °C incubation conditions. After 48 h, the log_10_ CFU/wound was used to reflect the overall colonization of *M. caribbica* populations. Three replicates were included for each experiment.

### 2.4. The Adhesion of M. caribbica to A. alternata

#### 2.4.1. Microscopy Observation of *M. caribbica* and *A. alternata*

Microscopy of *M. caribbica* adhesion to *A. alternata* was performed based on the method of Liu et al. [9]. A 10 μL suspension of *A. alternata* (1 × 10^6^ spores/mL) was used to inoculate slides containing small pieces of the PDA medium (2 cm × 2 cm) and then incubated at 25 °C until a large number of visible mycelia were formed (approximately 24 h). The hyphal surface of *A. alternata* was infected with an equal amount of *M. caribbica* cell suspension (1 × 10^6^ cells/mL). The mixed cultures were cultivated for 24 and 48 h and then rinsed with SDW. The state of the mixed cultures was observed using a light microscope (Olympus, Tokyo, Japan).

#### 2.4.2. SEM Observation of *M. caribbica* and *A. alternata* in the Jujube Wound

The interactions of *M. caribbica* and *A. alternata* in jujube wounds were observed as described by Chen et al. [32]. The wound preparation, microbial inoculation, and subsequent storage of jujube fruit were conducted following the steps in Section 2.2. Two days after storage, the tissue (5 mm × 3 mm × 3 mm) was cut from the jujube wounds to prepare electron microscopy samples by using a sterile scalpel. Sample tissues were fixed overnight at 4 °C with electron microscopy fixative (containing 2.5% glutaraldehyde). Subsequently, sample tissues were impregnated with gradient ethanol to remove water, followed by soaking in gradient tert-butanol to displace the ethanol. The sample tissues were dried at 60 °C for 2.5 h using a vacuum desiccator (DZF-6051, China). Tissue surfaces were gold-plated and observed under a scanning electron microscope (SEM, Phenom Pro, Phenom World, Eindhoven, The Netherlands).

### 2.5. Detection of Biofilms Formed by M. caribbica

Biofilm formation by *M. caribbica* was evaluated using the assay mentioned by Parafati et al. [33]. Fresh *M. caribbica* cell suspension was added to YNB (Hopebio, Shandong, China) containing glucose at 100 mmol/L, with an adjusted concentration of 1 × 10^7^ cells/mL. YNB without yeast addition was used as a control. One hundred microliters of *M. caribbica* cell suspension was incubated in polystyrene plates at 28 °C for 3, 8, 24, 48, and 72 h at 75 rpm. After incubation for the corresponding time periods, the polyethylene plates were removed, and the incubation solution was washed with PBS (pH 7.2). Then, an equal amount of 0.4% crystalline violet solution was added for 45 min of staining. Following staining, the unadsorbed stain was washed away with PBS, and 200 μL of 95% ethanol was added for decolorization. The processing of decolorization was completed after 45 min, and the decolorized solution absorbance value measured at 590 nm was used to represent the biofilm formation ability. Each measurement was replicated three times, and the experiment was conducted in triplicate.

### 2.6. Effect of Phenylethanol on Biofilm Formation of M. caribbica

#### 2.6.1. Biofilm Formation of *M. caribbica* in CM Medium

The conditioned medium (CM) was prepared according to Albuquerque et al. [34]. *M. caribbica* was incubated in NYDB in a shaker incubator for 5 days (200 rpm, 28 °C). After centrifugation of the *M. caribbica* fermentation broth, the collected supernatant liquor was filtered through a 0.22 μm microporous membrane to obtain the CM medium. The cultured *M. caribbica* cells were suspended in YNB containing different levels of CM (final concentrations of 0%, 5%, 25%, 50%, 75%, and 100%) and adjusted to 1 × 10^7^ cells/mL as the final concentration of *M. caribbica* cells. The biofilm measurements were performed following the steps in Section 2.5.

#### 2.6.2. Evaluation of Phenylethanol Production from *M. caribbica* by HPLC

The phenylethanol concentration of CM was measured by HPLC according to the method of Lei et al. [18] with slight modifications. The CM medium was obtained following the treatment outlined in Section 2.6.1 and tested immediately. The experimental setup was as follows: a C_18_ column and a 260 nm UV detector at 30 °C. The detection of the CM medium was performed at a rate of 0.7 mL/min with a 20 μL injection volume. The liquids used were 0.6% acetic acid solution as mobile phase A and methanol as mobile phase B. 

#### 2.6.3. Effect of Phenylethanol on Biofilm Formation by *M. caribbica*

The cultured *M. caribbica* cells were suspended in a YNB medium containing various phenylethanol concentrations (final concentrations of 0, 1, 2, and 4 mmol/L) to adjust the *M. caribbica* cell concentration to 1 × 10^7^ cells/mL. Biofilm formation assays were performed as outlined in Section 2.5.

### 2.7. Influence of Phe on the Biocontrol Efficiency of M. caribbica

#### 2.7.1. Effect of Phe on Phenylethanol Production of *M. caribbica*

The cultured *M. caribbica* cells were suspended in NYDB with various concentrations of Phe (final concentrations of 0, 1, and 8 mmol/L) to achieve a *M. caribbica* cell concentration of 1 × 10^7^ cells/mL and were cultured on a shaker (28 °C, 200 rpm) for 5 days. The content of phenylethanol was tested following the treatment outlined in Section 2.6.2.

#### 2.7.2. Effect of Phe on Biofilm Formation of *M. caribbica*

Freshly cultured yeast cells were added to the YNB medium containing different concentrations of Phe (final concentrations of 0, 1, and 8 mmol/L) and reached a concentration of 1 × 10^7^ *M. caribbica* cells/mL. The biofilm assay was performed as outlined in Section 2.5.

The state of *M. caribbica* in jujube wounds was observed by SEM. Fresh *M. caribbica* cells were inoculated in NYDB medium containing various concentrations of Phe (final concentrations of 0, 1, and 8 mmol/L). After 16 h of incubation, *M. caribbica* cells from the different treatment groups were collected. Jujube fruit and sample tissues were prepared according to Section 2.4.2. The *M. caribbica* cell suspension (20 μL; 1 × 10^8^ cells/mL) from each Phe treatment group was injected into the jujube wounds, with SDW inoculated as a control. The jujube fruits were stored for 2 days and then sampled for observation.

#### 2.7.3. Effect of Phe on the Biocontrol Assay of *M. caribbica*

The *M. caribbica* cell suspension obtained from each Phe treatment group was used to inoculate jujube fruit wounds prepared as described in Section 2.7.2. Jujubes inoculated with SDW served as a control. After 4 h, an additional suspension of 10 μL *A. alternata* spores was injected into each wound. After the jujube fruits were air-dried, they were stored in plastic baskets that maintained a stable temperature and humidity (25 °C, 90%). The DI and LD were recorded as described in Section 2.2. Each treatment was performed three times, with 10 fruits each time, and the experiment was replicated twice.

### 2.8. Statistical Analysis

SPSS 26.0 (SPSS Inc., Chicago, IL, USA) was used to conduct the data analysis for this study. The independent samples’ *t*-test and Duncan’s multiple comparison method were used for the ANOVA, and the statistical significance was denoted by *p* < 0.05.

## 3. Results

### 3.1. Efficiency of M. caribbica against Jujube Black Spot Rot

**Figure 1:**
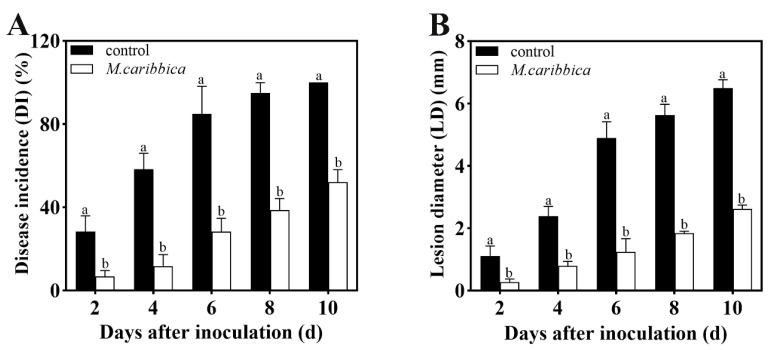
The biocontrol effect of *M. caribbica* inhibits *A. alternata* growth in jujube fruit. The (A) DI and (B) LD of jujube fruit were measured after inoculation at 25 °C. Standard errors of the means are indicated using vertical bars, and significant differences are denoted with different letters (*p* < 0.05).

*In vivo* experiments illustrate that the *M. caribbica* treatment dramatically prevented the rate of *A. alternata* infection in the jujube fruit (Figure 1). After storage at 25 °C for 10 days, the DI and LD of the jujube fruit were decreased by 48.0% and 59.7%, respectively, compared with the control. The rate of LD increase in *M. caribbica*-treated jujube fruit was much slower than that in the control for the entire storage period. This result demonstrated that *M. caribbica* had good biological control of jujube black spot rot.

### 3.2. Colonization of Jujube Wounds by M. caribbica

**Figure 2:**
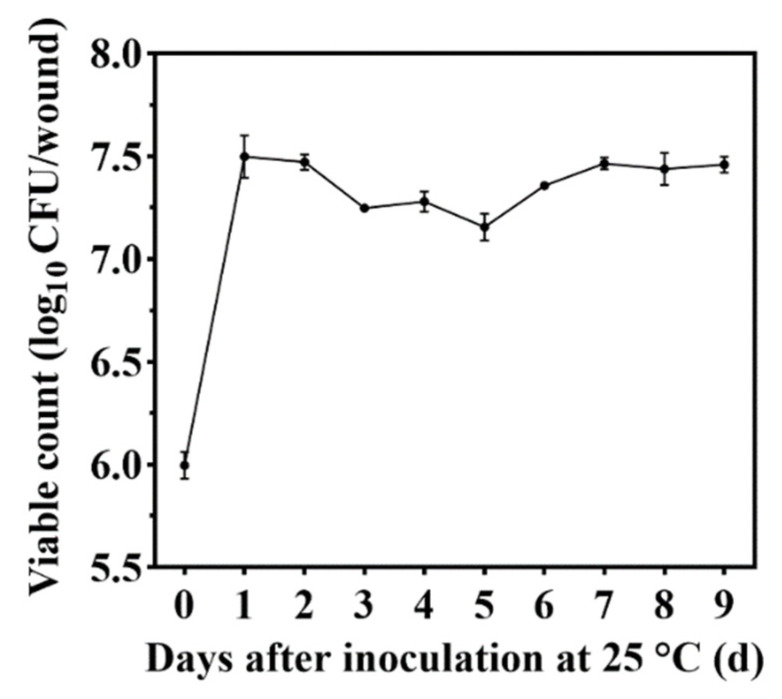
Colonization status of *M. caribbica* on jujube wounds. Vertical bars denote the standard error of the mean.

*M. caribbica* colonized jujube fruit wounds and grew rapidly under 25 °C storage conditions. This demonstrated the colonization changes of *M. caribbica* in jujube wounds over the entire storage period (Figure 2). The population of *M. caribbica* increased dramatically in the first 24 h, with the number increasing from an initial 5.99 to 7.50 log_10_ CFU/wound and then approaching a steady state. The viable count of *M. caribbica* was 7.46 log_10_ CFU/wound, which was a significant increase compared with the preliminary viable count after a 9-day storage period. These results indicated that *M. caribbica* had excellent colonization ability.

### 3.3. Adherence of M. caribbica to A. alternata

#### 3.3.1. In Vitro Interaction Study between *M. caribbica* and *A. alternata*

The mixed cultures of *M. caribbica* and *A. alternata* on slides were observed by microscopy. After rinsing the cocultures with SDW for 30 s, *M. caribbica* was still able to adhere tightly to the *A. alternata* hyphae, indicating an adhesive effect (Figure S1). However, no breakage or deformation of *A. alternata* hyphae was observed, indicating that *M. caribbica* had no parasitic effect on *A. alternata*.

#### 3.3.2. SEM Observation of *M. caribbica* and *A. alternata* on Jujube Wounds

**Figure 3:**
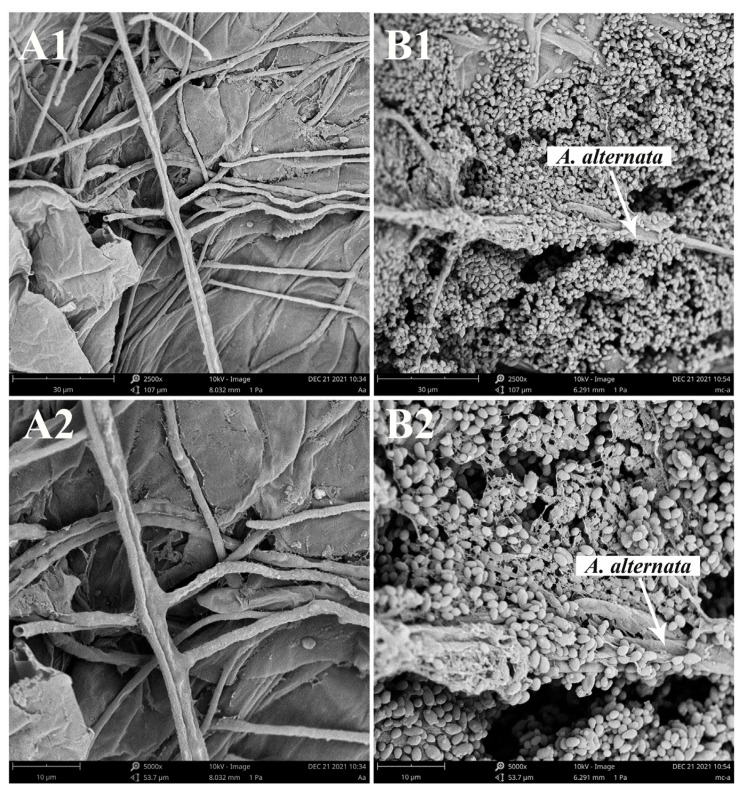
SEM of jujube wound tissues 48 h after inoculation. Wounds were incubated with (A) *A. alternata*, (B) *M. caribbica,* and *A. alternata*; (A1,B1): magnification of 2500×; (A2,B2): magnification of 5000×.

*A. alternata* alone multiplied on the wounds of jujube fruit with a large amount of hyphal production, as shown in Figure 3A. In contrast, inoculation with *M. caribbica* reduced the hyphal production of *A. alternata*. In addition, *M. caribbica* was observed to adhere closely to the hyphae of *A. alternata*, effectively reducing the physical space available for *A. alternata* growth (Figure 3B). The rapid colonization ability of *M. caribbica* at the wounds exerted strong spatial competition pressure on *A. alternata*, further inhibiting its infestation of the jujube fruit.

### 3.4. Biofilm-Forming Ability of M. caribbica

**Figure 4:**
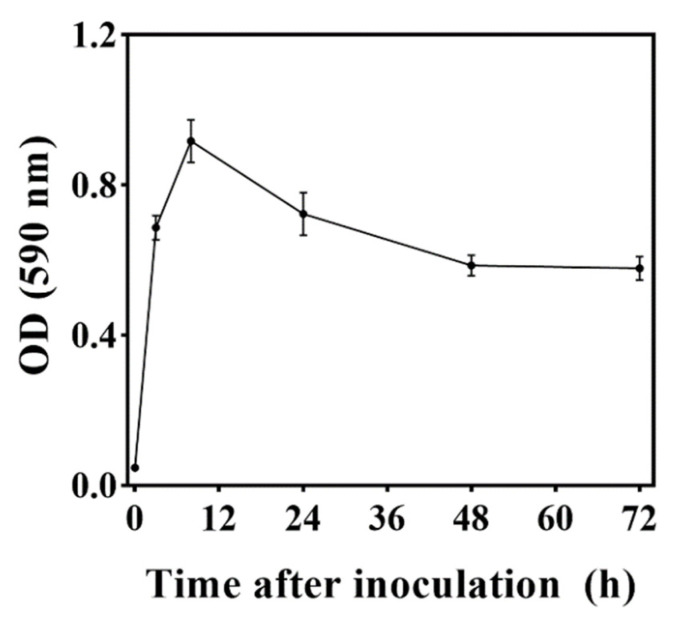
Biofilm formation ability on *M. caribbica* stained with crystal violet. The error bar reflects the standard deviation of the mean.

The OD_590_ value was used to report the biofilm formation ability of *M. caribbica*. *M. caribbica* exhibited high adherence to polystyrene plates and remained stably adherent to the plates after repeated washing. As shown in Figure 4, after 3 h of culture, *M. caribbica* could adhere to the polystyrene plates with an OD_590_ value of 0.67. It reached the maximum value (OD_590_ was 0.92) after 8 h of culture and then began to decrease. After 48 and 72 h of culture, the OD_590_ showed no significant change compared with 3 h but still maintained a high biofilm formation ability.

### 3.5. The Mechanism of Biofilm Formation in M. caribbica

#### 3.5.1. Development of *M. caribbica* Biofilm by CM Medium

**Figure 5:**
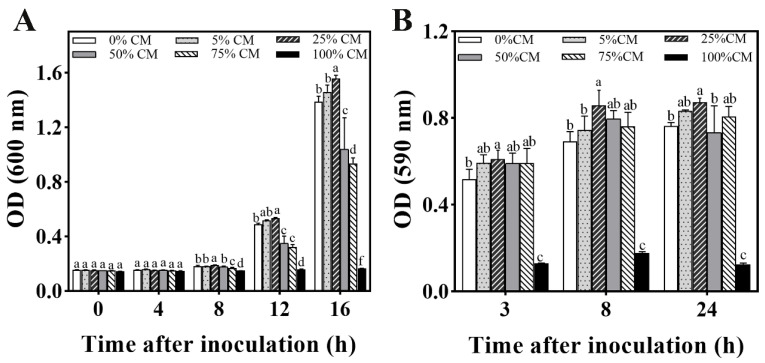
Effect of various concentrations of CM on (A) cell growth and (B) biofilm formation. Error bars indicate the standard deviation, and different letters indicate significant differences (*p* < 0.05).

The efficacy of CM on *M. caribbica* cell growth and biofilm development is shown in Figure 5. The 25% CM medium significantly stimulated the growth of *M. caribbica* during 8–16 h of incubation (Figure 5A). According to the OD_590_ value, the 25% CM medium significantly promoted the formation of *M. caribbica* biofilms during the culture time (Figure 5B). This result suggests that the presence of a substance in the CM medium can act as a QSM, affecting the biofilm development of *M. caribbica*. In contrast, the 100% CM medium was unfavorable for the growth of *M. caribbica* biofilms. The possible reason for this is that the 100% CM medium has few of the nutrients required for yeast and cannot sustain its normal growth.

#### 3.5.2. Phenylethanol Determination in CM Medium by HPLC

The presence of phenylethanol in the CM medium was measured by HPLC, as shown in Figure S2. The CM medium sample was examined, and a retention peak time similar to that of the phenylethanol standard was observed (Figure S2A,B), while the corresponding peak did not appear in the NYDB sample without the yeast addition (Figure S2C). This result suggests that phenylethanol is one of the metabolites of *M. caribbica* that is secreted into the medium.

#### 3.5.3. Biofilm Formation in *M. caribbica* Induced by Phenylethanol

**Figure 6:**
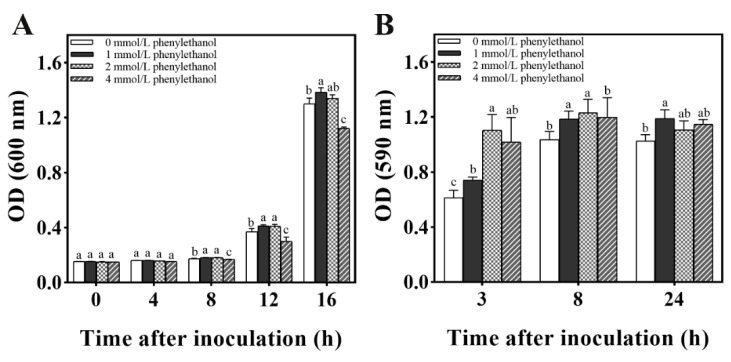
Effect of various concentrations of phenylethanol on (A) cell growth and (B) biofilm formation. Error bars indicate the standard deviation, and different letters indicate significant differences (*p* < 0.05).

As shown in Figure 6, different concentrations of phenylethanol were demonstrated to have an effect on the formation of *M. caribbica* biofilms. During the initial 3 h incubation time, 1, 2, and 4 mmol/L of phenylethanol significantly enhanced the biofilm of *M. caribbica*. At 8 h of incubation, 1 and 2 mmol/L phenylethanol significantly enhanced the biofilm formation of *M. caribbica*. At 24 h of incubation, 1 mmol/L phenylethanol significantly enhanced biofilm formation. Phenylethanol (1 mmol/L) was shown to promote biofilm formation throughout the culture time, and it significantly enhanced the growth of yeast during the incubation time of 8 to 16 h. This result suggested that phenylethanol is a QSM that influences the biofilm development of *M. caribbica*.

### 3.6. Influence of Phe on the Biocontrol Efficiency of M. caribbica

#### 3.6.1. Efficacy of Phe on Phenylethanol Production in *M. caribbica*

**Figure 7:**
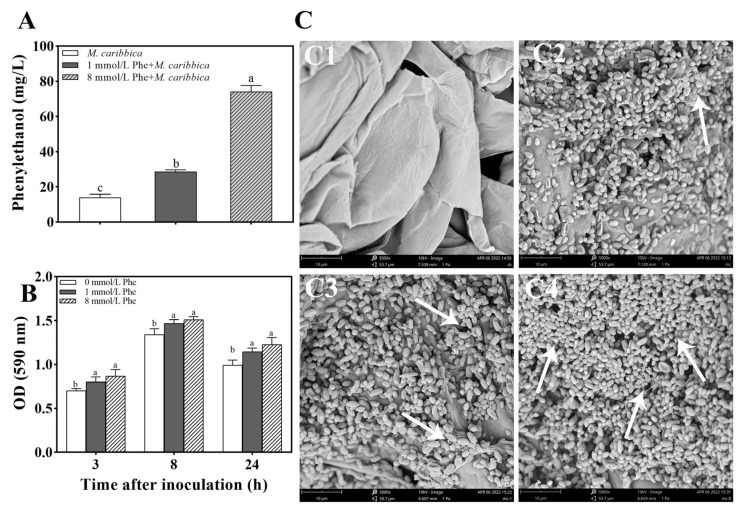
Effect of Phe on phenylethanol production and biofilm formation of *M. caribbica*. (A) Influence of different Phe concentrations on the phenylethanol content produced by *M. caribbica*. The vertical bar denotes the standard deviation of the mean. (B) Influence of different Phe concentrations on biofilm formation of *M. caribbica.* (C) SEM of jujube wound tissues 48 h after inoculation. Wounds were incubated with (C1) SDW, (C2) *M. caribbica*, (C3) *M. caribbica* obtained from NYDB medium with 1 mmol/L Phe, and (C4) *M. caribbica* obtained from NYDB medium with 8 mmol/L Phe. (C1–C4): magnification of 5000×. White arrows indicate the extracellular matrix secreted by *M.caribbica*. Error bars indicate the standard deviation, and different letters indicate significant differences (*p* < 0.05).

The content of phenylethanol in the CM medium was influenced by the addition of Phe. The high concentration of Phe was beneficial for the production of phenylethanol. The content of phenylethanol in CM supplemented with 8 mmol/L Phe was significantly higher than that in CM supplemented with 1 mmol/L Phe and CM without Phe (Figure 7A). The results indicate that the addition of Phe can increase the amount of phenylethanol produced by *M. caribbica*.

#### 3.6.2. Induction of Biofilm Formation in *M. caribbica* by Phe

The effect of various concentrations of Phe on the formation of biofilms by *M. caribbica* is depicted in Figure 7B. At 3 h of culture, Phe demonstrated the ability to increase the OD_590_ value compared with the group with no Phe addition. The addition of 1 and 8 mmol/L Phe significantly promoted the biofilm formation of *M. caribbica* during 3–24 h of incubation. The above results indicate that Phe enhances the biofilm formation of *M. caribbica*.

The state of *M. caribbica* in the jujube wounds is shown in Figure 7C2–C4. As seen in the scanning electron micrographs, secretion of the yeast extracellular matrix was observed in all samples except the control. *M. caribbica* treated with Phe secreted more of the extracellular matrix and aggregated to a greater extent between yeast cells, while yeast untreated with Phe showed less extracellular matrix secretion. This suggests that Phe promotes the formation of *M. caribbica* biofilms and adhesion in jujube wounds.

#### 3.6.3. Enhancement of Biocontrol Performance in *M. caribbica* by Phe

**Figure 8:**
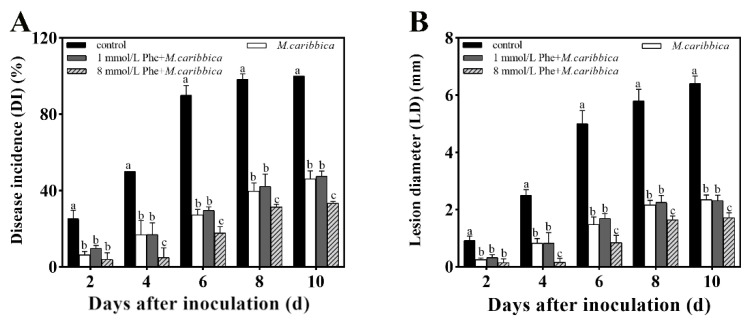
The effect of different concentrations of Phe on the biocontrol of jujube black spot rot by *M. caribbica*. The DI (A) and LD (B) were recorded for jujube black spot rot in fruit stored at 25 °C for 10 d. Error bars indicate the standard deviation, and different letters indicate significant differences (*p* < 0.05).

The efficacy of the Phe addition to the NYDB broth of *M. caribbica* in reducing jujube black spot rot is shown in Figure 8. Each *M. caribbica*-treated group significantly inhibited the growth of *A. alternata* in jujube fruit compared with the uninoculated yeast control. After a 10-day storage at 25 °C, *M. caribbica* cultured in NYDB broth with 8 mmol/L Phe showed the best efficacy in controlling black spot rot, as the DI and LD of the jujube fruits were significantly reduced by 12.67% and 21.75%, respectively, compared with those of the group treated with only *M. caribbica*. The results indicate that 8 mmol/L Phe significantly improves the biocontrol efficiency of *M. caribbica* against *A. alternata* in jujube fruit.

## 4. Discussion

Antagonistic yeast has proven to be a useful biocontrol tool for postharvest fungal diseases, is perceived as an appropriate alternative to chemical fungicides, and has an important role in biological control [35]. The capacity to target numerous pathogens in fruit is one of the criteria for being an ideal biocontrol agent [36]. The yeast *M. caribbica* has been reported to control postharvest anthracnose on mango and papaya fruit caused by *C. gloeosporioides* [37,38]. In our research, *M. caribbica* significantly reduced the fungal infection caused by the artificial inoculation of *A. alternata* in jujube fruits.

For our study, an important strategy used by *M. caribbica* against *A. alternata* was the competition for nutrients and available space. The excellent biocontrol performance of antagonistic yeast was associated with a high population density on the fruit [39]. *M. caribbica* rapidly colonized the jujube wounds after inoculation and maintained a stable population density throughout the entire duration of storage (Figure 2). The high population density of antagonistic yeast was conducive to biofilm coverage at the fruit wounds [40]. The mixed cultures of *M. caribbica* and *A. alternata* were observed using microscopy, and it was found that *M. caribbica* was not parasitic to *A. alternata* (Figure S1). The hyphae of *A. alternata* did not show deformation or breakage due to the attachment of *M. caribbica*, as observed by SEM. However, we found that *M. caribbica* firmly adhered to the surface of *A. alternata* hyphae (Figure S1). Around jujube wounds, the extracellular matrix encasing encapsulating yeast, as well as the secretion of aggregated yeast cells, were visible (Figure 3B). This indicated that *M. caribbica* competed for nutrient factors in the fruit wounds to rapidly multiply and form biofilms, occupying space in the wound sites to suppress *A*. *alternata* growth. Through in vitro experiments, *M. caribbica* was found to adhere tightly to polystyrene plates, demonstrating its strong biofilm formation ability (Figure 4).

Biofilm formation is regulated by QS, and QSMs have essential functions in biofilm development [41]. It was reported that QSMs should follow a density-dependent accumulation during microbial growth, and thus be able to induce a coordinated population response after reaching a specific concentration. Moreover, QSMs are able to reproduce the QS phenotype by the exogenous addition of substances [42]. In our study, it was demonstrated that QS was involved in regulating the formation of *M. caribbica* biofilms, and the 25% CM medium significantly improved the biofilm growth of *M. caribbica* (Figure 5B). This also suggests that it is possible for some metabolites in the CM medium to act as QSMs of *M. caribbica*. The major volatile metabolic organisms reported for *M. caribbica* include 1-butanol, 3-methyl, phenylethanol, and ethyl acetate [43,44]. Phenylethanol is a reportedly important communication molecule that regulates the population behavior and biofilm formation of *S. cerevisiae* and *D. hansenii* [45,46]. In our study, phenylethanol, which is the secondary metabolite of *M. caribbica*, was assayed in the CM medium by HPLC (Figure S2). Moreover, the formation of *M. caribbica* biofilms was enhanced by the exogenous addition of phenylethanol, which indicated that phenylethanol acted as a QSM of *M. caribbica*.

The synthesis of phenylethanol in microorganisms is based on the precursor substance Phe. Interestingly, we have found that some yeasts are able to convert Phe into phenylethanol: a method commonly used in the industrial production of natural phenylethanol [47]. *S. cerevisiae*, for example, can ferment Phe via the Ehrlich pathway to produce more phenylethanol [48]. In the present research, Phe stimulated the biofilm formation of *M. caribbica* and contributed to more extracellular matrix secretion by *M. caribbica*, which assisted in the adhesion to jujube fruit wounds (Figure 7C). HPLC revealed that Phe could stimulate *M. caribbica* to secrete more phenylethanol into the culture. Moreover, a positive correlation was found between Phe, *M. caribbica* biofilm formation, and phenylethanol content (Figure S3). This suggests that Phe can influence the biofilm formation of *M. caribbica* at the level of phenylethanol. It is interesting to note that some QSM-producing precursors, such as amino acids, also affect the morphological structure and biofilm formation of yeast. Indeed, 2-Methyl-1-butanol is one of the QSMs of the dimorphic fungus *Ophiostoma ulmi*, which can affect its morphological transformation, while isoleucine (a precursor of 2-methyl-1-butanol) has analogous effects on the morphological transformation of *O. ulmi* [49]. Nitrogen sources can alter the morphology of *Pichia fermentans*, and methionine, valine, and Phe can induce pseudohyphal morphology [50].

In the in vivo experiment, *M. caribbica* treated with 8 mmol/L Phe performed the best biocontrol effect against black spot rot, and the addition of Phe was negatively correlated with jujube disease incidence (Figure S3). A negative correlation was also found between the formation of *M. caribbica* biofilm and fruit diseases. This result suggested that Phe had a positive impact on the effectiveness of *M. caribbica* as a biocontrol agent, which greatly depends on its biofilm enhancement. Notably, in a study conducted by Klein and Kupper [51], 1% ammonium sulfate stimulated the production of *Aureobasidium pullulans* biofilms, thereby increasing the inhibitory efficiency against *G. citri-aurantii* and effectively controlling citrus decay. Additionally, Wang’s study showed that the biocontrol potency of *M. citriensis* against citrus sour rot was enhanced by arginine treatment by promoting its adhesion to citrus fruit and inducing an increase in its oxidative stress tolerance [52]. These studies emphasized that the enhancement of antagonistic yeast biofilms is associated with improving their biocontrol efficacy.

## 5. Conclusions

In the current study, our results demonstrated that *M. caribbica* exhibited excellent efficacy in the control of jujube black spot rot. *M. caribbica* can rapidly colonize fruit wounds and has a potent biofilm-forming ability. The formation of *M. caribbica* biofilms was influenced by QSMs. Phenylethanol, as a QSM, regulated the biofilm growth of *M. caribbica*. In addition, Phe, as a precursor substance of phenylethanol, promoted the formation of *M. caribbica* biofilms by increasing the content of phenylethanol, thus enhancing the ability of *M. caribbica* to control black spot rot on the jujube. The present results are of great significance for enhancing the bioprotective efficacy of antagonistic yeast, and also provide a promising strategy for the control of postharvest disease in jujube fruits.

## Data Availability

All the data supporting the findings of this study are included in this article.

## Supplementary Materials

The following supporting information can be downloaded at: https://www.mdpi.com/article/10.3390/jof8121313/s1, Figure S1: Interaction between *M. caribbica* and *A. alternata* for (A1,B1) 24 h/(A2,B2) 48 h after incubation. The *A. alternata* hyphae alone were used as a control. A–B: magnification of 1000×. Figure S2: HPLC analysis of phenylethanol in CM (A–C). (A) CM medium, (B) Standard: commercial phe-nylethanol, and (C) NYDB medium. Figure S3: Pearson’s correlation analysis among Phe, phenylethanol content, biofilm, and disease incidence. Each square color scale represents the correlation coefficient. * indicates significant correlation (*p*< 0.05), ** indicates highly significant correlation (*p* < 0.01).
