# Supplementary material for: Phenylalanine Promotes Biofilm Formation of Meyerozyma caribbica to Improve Biocontrol Efficacy against Jujube Black Spot Rot
Source: J Fungi (Basel). 2022 Dec 17;8(12):1313. doi: 10.3390/jof8121313 (PMC9786301; doi:10.3390/jof8121313)
Supplement: Supplementary file 1 [file jof-08-01313-s001.zip › jof-2084254-supplementary.pdf]

Journal of
Fungi

MDPI

Article

Phenylalanine Promotes Biofilm Formation of *Meyerozyma caribbica* to Improve Biocontrol Efficacy against Jujube Black Spot Rot

Qian Deng [1], Xingmeng Lei [1], Hongyan Zhang [1], Lili Deng [1,2], Lanhua Yi [1,2] and Kaifang Zeng [1,2,3,*]

[1] College of Food Science, Southwest University, Chongqing 400715, China
[2] Food Storage and Logistics Research Center, Southwest University, Chongqing 400715, China
[3] Chongqing Key Laboratory of Speciality Food Co-Built by Sichuan and Chongqing, Chongqing 400715, China
* Correspondence: zengkaifang@hotmail.com

Citation: Deng, Q.; Lei, X.; Zhang, H.; Deng, L.; Yi, L.; Zeng, K. Phenylalanine Promotes Biofilm Formation of *Meyerozyma caribbica* to Improve Biocontrol Efficacy against Jujube Black Spot Rot. *J. Fungi* **2022**, *8*, 1313. https://doi.org/10.3390/jof8121313

Academic Editors: Nengguo Tao and Xiaoli Tan

Received: 23 November 2022
Accepted: 13 December 2022
Published: 17 December 2022

Publisher's Note: MDPI stays neutral with regard to jurisdictional claims in published maps and institutional affiliations.

Copyright: © 2022 by the authors. Licensee MDPI, Basel, Switzerland. This article is an open access article distributed under the terms and conditions of the Creative Commons Attribution (CC BY) license (https://creativecommons.org/licenses/by/4.0/).

Supplementary Materials

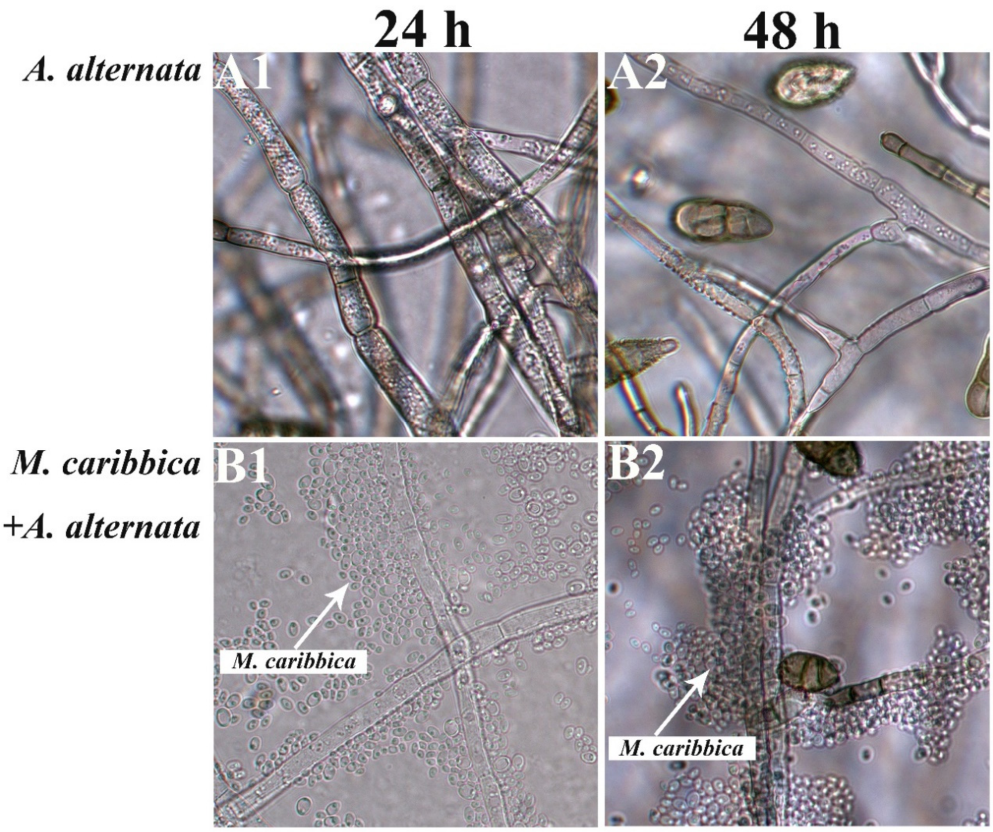

Figure S1. Interaction between *M. caribbica* and *A. alternata* for (A1,B1) 24 h/(A2,B2) 48 h after incubation. The *A. alternata* hyphae alone were used as a control. A-B: magnification of 1000×.

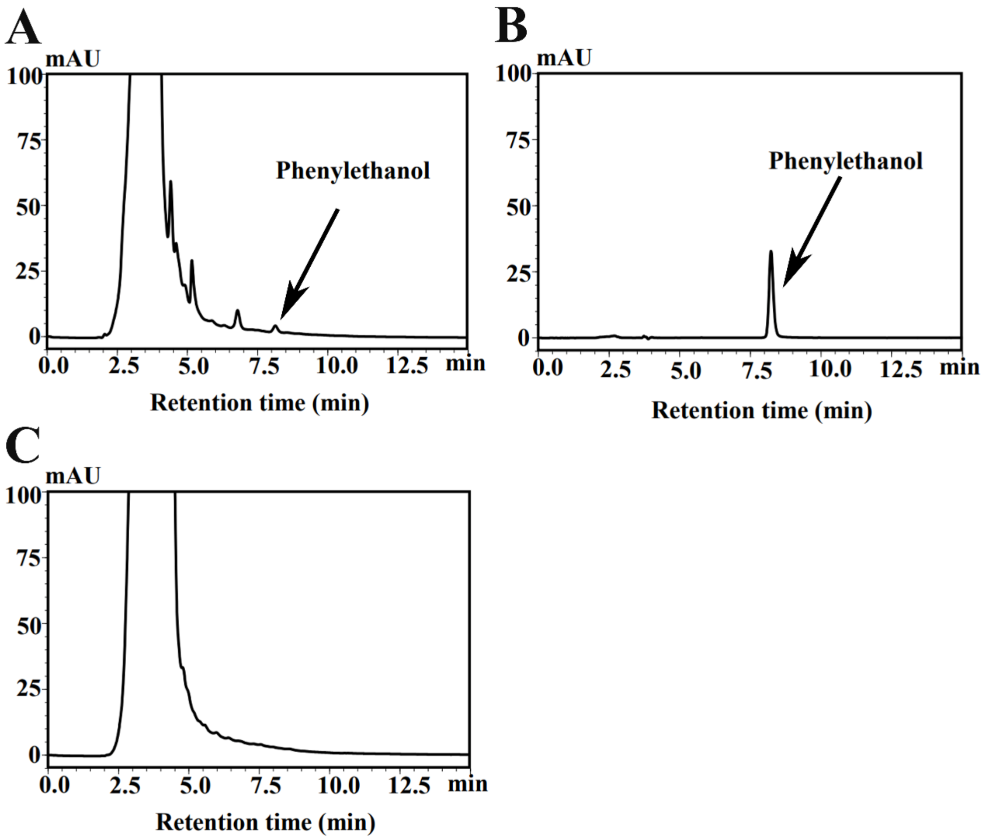

Figure S2. HPLC analysis of phenylethanol in CM (A–C). (A) CM medium, (B) Standard: commercial phenylethanol, and (C) NYDB medium.

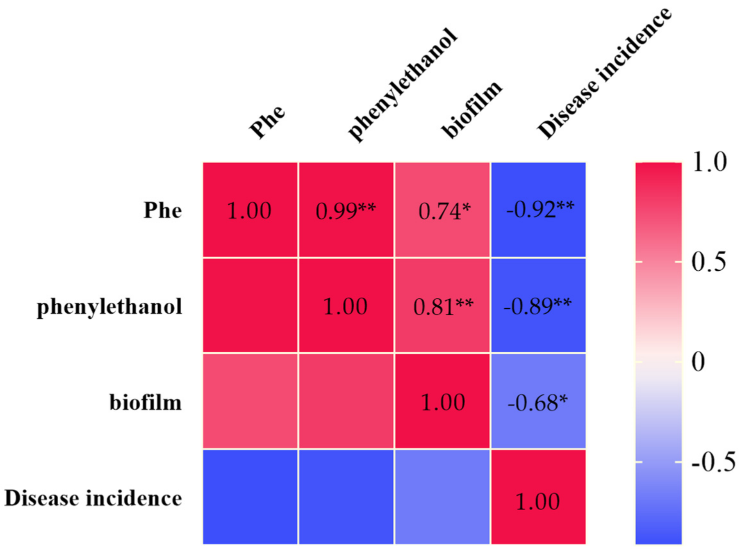

Figure S3. Pearson's correlation analysis among Phe, phenylethanol content, biofilm, and disease incidence. Each square color scale represents the correlation coefficient. * indicates significant correlation ($p < 0.05$), ** indicates highly significant correlation ($p < 0.01$).
